# Spontaneous Subdural Empyema Following a High-Parasitemia *Falciparum* Infection in a 58-Year-Old Female From a Malaria-Endemic Region: A Case Report

**DOI:** 10.1177/2324709616666567

**Published:** 2016-08-30

**Authors:** Pedro Pallangyo, Frederick Lyimo, Paulina Nicholaus, Ulimbakisya Kain, Mohamed Janabi

**Affiliations:** 1 Jakaya Kikwete Cardiac Institute, Dar es Salaam, Tanzania; 2 Muhimbili National Hospital, Dar es Salaam, Tanzania

**Keywords:** spontaneous subdural empyema, falciparum malaria, severe malaria, cerebral malaria, *Plasmodium falciparum*, sub-Saharan Africa

## Abstract

Malaria remains a significant public health problem of the tropical world. *Falciparum* malaria is most prevalent in the sub-Saharan African region, which harbors about 90% of all malaria cases and fatalities globally. Infection by the *falciparum* species often manifests with a spectrum of multi-organ complications (eg, cerebral malaria), some of which are life-threatening. Spontaneous subdural empyema is a very rare complication of cerebral malaria that portends a very poor prognosis unless diagnosed and treated promptly. We report a case of spontaneous subdural empyema in a 58-year-old woman from Tanzania who presented with high-grade fever, decreased urine output, and altered sensorium.

## Introduction

Malaria is a mosquito-borne disease of public health importance that threatens nearly one half of the global population.^1^ Sub-Saharan African region, which harbored about 90% of all malaria cases and deaths in 2015, is disproportionately affected.^1^ *Falciparum* malaria is known for its myriad clinical presentations ranging from self-limiting to life-threatening. Spontaneous subdural empyema (SDE), which refers to a collection of pus between the dura and arachnoid mater, is one of the unusual but highly fatal complication of *Plasmodium falciparum* malaria.^2^

Diagnosing spontaneous SDE in patients with cerebral malaria is often a dilemma, and if diagnosis and management is delayed, the risk of long-term neurological sequelae and death is very high.^3,4^ In this antibiotic era, SDE-related mortality is reportedly between 14% and 18%; however, in underprivileged nations mortality rate approaches 100%, similar to the pre-antibiotic era.^5^ Moreover, clinical presentation during admission is a good prognostic indicator, that is, the mortality rate is <10% in alert patients and rises to 75% in patients presenting with coma.^6^ We report a case of a 58-year-old woman from Tanzania who developed a spontaneous SDE following a high-parasitemia *falciparum* infection.

## Case Report

A 58-year-old woman was referred to us from a private facility with a 6-day history of altered sensorium and high-grade fever. She has history of systemic hypertension for 8 years with poor medication adherence. She was diagnosed with high-parasitemia *falciparum* malaria (blood slide for malaria parasite >1000/200 white blood cells), and the sequestered red cells contained the mature forms of the parasite (trophozoites and meronts). She was started on intravenous artesunate 240 mg at 0, 12, 24, and 48 hours, and intravenous clindamycin 600 mg twice daily for 3 days was also administered as per the current local guidelines for the management of severe *falciparum* malaria. However, due to clinical deterioration, she was referred to us on the fourth day of hospitalization in the private facility for expert management. There was no prior history of head injury or fall from a height, convulsions, or anticoagulant use. Furthermore, there was no history suggestive of recent sinusitis, rhinitis, or mastoiditis.

On admission, she was febrile (39.2°C) with a Glasgow Coma Score (GCS) of 9/15 (E_4_V_2_M_3_). Her blood pressure was 156/109 mm Hg, pulse rate was 116 beats/minute, random blood glucose was 12.2 mmol/L, and she weighed 90.3 kg. She had a reduced urine output (300 mL/24 hours) with elevation of urea (33.2 mmol/L; range = 1.8-7.1 mmol/L) and creatinine (442.3 µmol/L; range = 46-92 µmol/L). On her blood count, leukocyte concentration was 15.4 × 10^9^/L (range = 4.5-10.5 × 10^9^/L) with neutrophilia (75%), hemoglobin was 9.5g/dL (range = 12-16 g/dL), and she had thrombocytopenia (platelets 62.7 × 10^9^/L; range = 150-450 × 10^9^). In liver panel, she had elevated aspartate transaminase (203 IU/L; range = <20 IU/L) but normal alanine transaminase (38 IU/L), and both direct (26.0 µmol/L; range = <7 µmol/L) and total bilirubin (44.3 µmol/L; range = 5-17 µmol/L) were elevated. She tested negative for HIV and had normal electrolytes. Electrocardiogram revealed sinus tachycardia (rate 124) and ECHO showed mild left ventricular hypertrophy with good systolic function (ejection fraction 62%). Intravenous artesunate 240 mg was reinstated, and she received 3 units of platelet concentrate and was resumed on her antihypertensives (amlodipine, hydralazine, and furosemide). Hemodialysis was also started, and after 5 sessions, her renal functions returned to normal (creatinine 76.8 µmol/L; urine output 950 mL/24 hours); however, her altered mentation did not improve.

**Figure 1.**
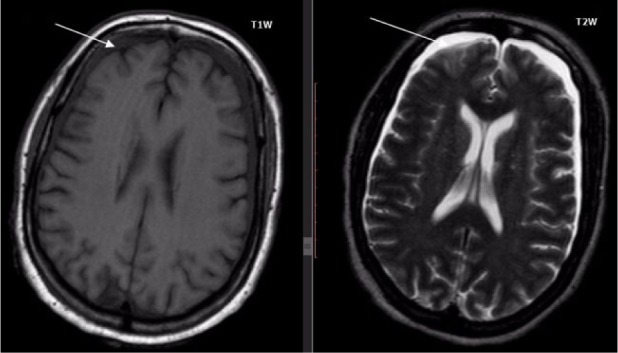
Brain MRI (T1W and T2W) showing bilaterally thin but widely spread subdural collection in the frontoparietal region.

**Figure 2.**
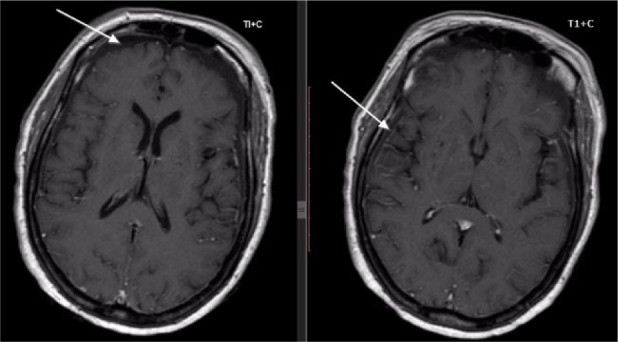
Brain MRI (T1+ C) showing bilaterally thin but widely spread subdural collection with peripheral meningeal enhancement in the frontoparietal region. Leptomeningeal enhancement is also seen.

A brain magnetic resonance imaging (MRI) was then ordered, which revealed a bilaterally thin but widely spread subdural collection in the frontoparietal region (Figures 1 and 2). A diagnosis of SDE was then reached and neurosurgeons were consulted promptly. The patient was started on intravenous meropenem, antiepileptic prophylaxis, and underwent a frontotemporal osteoplastic flap craniotomy in which thick pus was removed on dura mater opening. Analysis of the purulent fluid revealed pus cells with no organism. Twenty-four hours postoperative, GCS started improving, and she was afebrile (37.1°C) and had a fasting blood glucose of 3.9 mmol/L. On the fifth day postoperative, she started speaking comprehensively, and her postsurgery MRI and renal and liver functions were normal. Her postoperative blood slide for malaria parasite was negative. She was discharged home after a total of 21 days of hospitalization.

## Discussion

Severe *falciparum* malaria is associated with numerous complications, including cerebral malaria, pulmonary edema, acute renal failure, severe anemia, and metabolic acidosis. Although these complications may have variable presentations depending on age and geographical location, their potential for rapid development and progression to death remain high.^7^ In majority of patients with severe malaria such complications coexist or rather evolve rapidly and thus the presence of one of them should warrant both searching for others and aggressive management.

Despite its rarity, subdural empyema accounts for about a third of focal intracranial infections with frontal sinusitis as the leading etiology.^8^ Hemorrhagic complications including subarachnoid hemorrhage, cerebral hemorrhage, subdural empyema, and subdural hematoma are potential but extremely rare sequelae of falciparum malaria.^9-13^ Furthermore, although focal neurological signs are not a typical feature in malaria presentation, its presence should raise an index of suspicion for a spontaneous SDE diagnosis. On extensive literature search we did not find any case of bilateral SDE associated with *falciparum* malaria. Nevertheless, prompt diagnosis coupled by initiation of broad-spectrum antibiotics is one single indicator of high importance for favorable outcome in SDE. In conclusion, the presence of focal neurological deficits in patients with severe *falciparum* malaria should be regarded as a neurosurgical emergency unless brain imaging indicates otherwise.
